# Hormone Therapy Reduces Recurrence in Stage II-IV Uterine Low-Grade Endometrial Stromal Sarcomas: A Retrospective Cohort Study

**DOI:** 10.3389/fonc.2022.922757

**Published:** 2022-06-28

**Authors:** Xiaodi Huang, Peng Peng

**Affiliations:** Department of Obstetrics and Gynecology, Peking Union Medical College Hospital, Chinese Academy of Medical Sciences and Peking Union Medical College, National Clinical Research Center for Obstetric and Gynecologic Diseases, Beijing, China

**Keywords:** low-grade endometrial stromal sarcoma, hormone therapy, Recurrence, Disease free survival, Fertility-sparing

## Abstract

Low-grade endometrial stromal sarcoma (LG-ESS) is a rare and indolent malignancy. Hormone therapy has been reported as an adjuvant treatment for LG-ESS, although its effectiveness is controversial. Here we aimed to investigate the effects of postoperative hormone therapy on recurrence in patients with uterine LG-ESS. Between January 2010 and December 2019, a total of 152 patients (23 with and 129 without fertility-sparing) with a diagnosis of primary uterine LG-ESS confirmed by pathologists were enrolled in this study. In the cohort without fertility-sparing, 22 (17.7%) patients had recurrence, and the median disease-free survival (DFS) was 47 (2-130) months; only one of these patients died of LG-ESS. No significant difference was found in recurrence between the groups with and without hormone therapy (p=0.802). However, subgroup analysis showed that hormone therapy decreased the recurrence rate in stage II-IV (p=0.001, HR 0.144, 95% CI: 0.038-0.548), but not in stage I disease (p=0.256). High-dose progestins notably reduced recurrence (p=0.012, HR 0.154, 95% CI: 0.036-0.660), whereas non-progestin therapy marginally influenced recurrence (p=0.054) compared with no hormone therapy in stage II-IV disease. Moreover, hormone therapy within 12 months was effective in reducing recurrence (p=0.038, HR 0.241, 95% CI: 0.063-0.922). Ovarian preservation (p=0.004, HR 6.250, 95% CI: 1.786-21.874) and negative expression of ER/PR (p=0.000, HR 23.249, 95% CI: 4.912-110.026) were high-risk factors for recurrence in patients without fertility-sparing. In the fertility-sparing cohort, 15 (65.2%) patients experienced recurrence, and the median DFS was 24 (3-107) months. Six patients successfully delivered healthy fetuses, and five received hormone therapy. Twelve patients finally accepted hysterectomy after repeated recurrence, and only two of them had given birth before surgery. Patients who received hormone therapy showed longer DFS, although this difference was not statistically significant (p=0.466). In conclusion, postoperative hormone therapy reduces recurrence in patients with stage II–IV uterine LG-ESS without fertility-sparing, and high-dose treatment with progestins within 12 months is recommended. Bilateral oophorectomy can also reduce the risk of recurrence. Patients with fertility-sparing have a high risk of recurrence and poor pregnancy outcomes, and hormone therapy may be a reasonable choice in postoperative management.

## Introduction

Endometrial stromal sarcoma (ESS) is a rare malignancy, accounting for approximately 20% of uterine sarcomas (1, 2). There are four categories of ESS: endometrial stromal nodule, low-grade endometrial stromal sarcoma (LG-ESS), high-grade endometrial stromal sarcoma (HG-ESS), and undifferentiated uterine sarcoma (UUS) (3). Among these, LG-ESS is the most common uterine stromal sarcoma (4). LG-ESS is an indolent disease, with a protracted interval to recurrence (2, 5). Hysterectomy is recommended in patients with LG-ESS, and ovarian preservation could be considered in premenopausal patients (2, 6). Due to the slow-growing nature LG-ESS, in most cases of early-stage disease (2, 7), fertility-sparing surgery is performed in patients with a desire to have children. The use of adjuvant hormone therapy, including high-dose progestins, aromatase inhibitors, and gonadotropin-releasing hormone agonists (GnRH-a), has been reported for the treatment because LG-ESS is considered as a hormone-dependent tumor (8–10). These different drugs work by different mechanisms. Progestins have an antioestrogenic effect and suppress stromal endometrial proliferation by binding to progesterone receptor (11). Besides, progestins also involve in cell cycle regulation by cyclin-dependent kinase (12). Aromatase inhibitors reduce estrogen levels by blocking aromatase activity in peripheral adipose and tumor tissue (13).GnRH-a suppress ovarian estrogen production by inhibiting the pituitary ovarian axis, leading to a “postmenopausal” status; moreover, GnRH-a may have an additive action by blocking the intra-tumoral GnRH receptor (11, 13). However, whether hormone therapy can reduce the recurrence of LG-ESS remains controversial (14, 15). Therefore, in this study, we aimed to investigate the impact of postoperative hormone therapy on recurrence in patients with uterine LG-ESS. In addition, patients with and without fertility-sparing were analyzed separately, to better understand the effects of hormone therapy.

## Materials and Methods

### Patients

This was a single-center retrospective cohort study. A total of 155 patients with primary uterine LG-ESS confirmed by pathologists at our hospital were enrolled, between January 2010 and December 2019. Among these patients, three with high-grade ESS elements in recurrent pathology were excluded; thus, a total of 152 patients were analyzed. The study was approved by the Institutional Review Board of Peking Union Medical College Hospital (No. S-K2016), and the requirement for informed consent was waived.

Patients were divided into two cohorts depending on whether they preserved fertility. In the cohort without fertility-sparing, all patients underwent hysterectomy with or without bilateral oophorectomy. In the fertility-sparing cohort, only resection of lesions was performed. The stage of LG-ESS was determined according to the 2009 International Federation of Gynecology and Obstetrics (FIGO) staging system. Postoperative hormone therapy included high-dose progestins [megestrol acetate (MA) 160-320 mg/day or medroxyprogesterone acetate (MPA) 250-500 mg/day)], letrozole (2.5 mg/day), and GnRH-a (3.75 mg/28 days). A levonorgestrel-releasing intrauterine device (LNG-IUD) was used in some fertility-sparing patients. Moreover, some patients received radiotherapy or chemotherapy after surgery.

### Follow-Up and Measure Outcomes

After surgery, the patients were regularly followed up at our hospital, or other local hospitals. Follow-up methods included pelvic examination, blood tests, abdominopelvic ultrasonography, annual chest X-ray, and annual CT of the chest, abdomen, and pelvic cavity. Excluding those lost to follow-up, all patients were followed up until May 2021 by telephone or outpatient visits. The primary outcome was disease-free survival (DFS), defined as the time from surgery to recurrence or the last follow-up visit, whichever occurred first. Only one patient died of LG-ESS; therefore, we did not calculate overall survival. In addition, we also explored high-risk factors for recurrence in the cohort without fertility-sparing.

### Statistical Analysis

All statistical analyses were performed using SPSS version 23.0. Survival analysis was performed using the Kaplan-Meier method and the log-rank test. Univariate and multivariate Cox regression analyses were used to analyze prognostic factors and estimate the hazard ratio (HR) with a 95% confidence interval (95% CI). GraphPad Prism version 9.3 was used to draw survival curves. Statistical significance was set at P < 0.05.

## Results

### Outcomes in the Cohort Without Fertility-Sparing

A total of 129 patients were enrolled in this cohort, and their epidemiological characteristics, treatment, and follow-up results are shown in **Table 1**. The median patient age was 43 (20–67) years, and the median body mass index (BMI) was 23.0 (17.2-37.3) kg/m^2^. The vast majority (93%) of patients were at premenopausal stage. Bilateral oophorectomy was performed in 80.6% (n = 104), whereas at least one ovary was preserved in 19.4% (n = 25) of patients. The tumor diameter was >5 cm in 60.5% of patients, and lymphovascular space involvement (LVSI) was positive in 34.9% of patients. Immunohistochemical staining for estrogen receptor/progesterone receptor (ER/PR) was positive in 76.7% (n=99), negative in 3.1% (n=4), and unknown in 20.2% (n=26) of patients. Only 18.6% of patients experienced elevated levels of serum CA125. According to the FIGO staging system, 69.8% (n=90) of patients had stage I and 30.2% (n=39) had stage II–IV disease. Post-surgery, 32 patients received radiotherapy whereas seven patients received chemotherapy. There were 75 patients that received postoperative hormone therapy. Among those, 53 received high-dose progestins, 13 received letrozole, and nine received GnRH-a or a combination of two drugs. In addition, 41.1% (n=53) of patients did not receive any hormone therapy. In terms of duration of hormone therapy, 44.0% (n=33) of patients were within 6 months, 26.7% (n=20) were between 6 and 12 months, and 28.0% (n=21) were over 12 months.

**Table 1:** Epidemiological characteristics, treatment, and follow-up of the cohort without fertility-sparing.

Parameters	Patients (n=129)
Ages at diagnosis (years), median (range)	43 (20–67)
Menopausal status, n (%)	
Premenopausal	120 (93)
Postmenopausal	9 (7)
BMI at diagnosis (kg/m^2^), median (range)	23.0 (17.2-37.3)
Bilateral oophorectomy, n (%)	
Yes	104 (80.6)
No	25 (19.4)
Diameter of tumor, n (%)	
≤5cm	32 (24.8)
>5cm	78 (60.5)
not reported	19 (14.7)
LVSI, n (%)	
Positive	45 (34.9)
Negative	84 (65.1)
Immunohistochemical staining of ER/PR, n (%)	
Both negative	4 (3.1)
ER and/or PR positive	99 (76.7)
Not reported	26 (20.2)
CA125 level, n (%)	
Always<35U/ml	100 (77.5)
Once elevated	24 (18.6)
Not reported	5 (3.9)
Stage, n (%)	
I	90 (69.8)
II	23 (17.8)
III	8 (6.2)
IV	8 (6.2)
Postoperative hormone therapy^*^, n (%)	
High-dose progestins	53 (41.1)
Letrozole	13 (10.1)
Others^**^	9 (7.0)
None	53 (41.1)
Not reported	1 (0.8)
Duration of hormone therapy, n (%)	
≤6 months	33/75 (44.0)
6-12 months	20/75 (26.7)
>12 months	21/75 (28.0)
Not reported	1/75 (1.3)
Radiotherapy	32 (24.8)
Chemotherapy	7 (5.4)
Recurrence after surgery, n (%)	22/124^#^ (17.7)
DFS (months), median (range)	47 (2–130)
Death of disease, n (%)	1/121 (0.8)
Follow-up time (months), median (range)	58 (6-135)
Loss to follow-up, n (%)	8 (6.2)

^*^One patient stopped progestin and one patient stopped letrozole because of elevated liver enzymes. One patient changed from progestin to letrozole because of weight gain of 16 kg within 6 months. ^**^Other therapies included GnRH-a and two drugs combination. ^#^Three patients had recurrence before they were lost to follow-up. BMI, body mass index; LVSI, lymphovascular space involvement; ER, estrogen receptor; PR, progesterone receptor; DFS, disease-free survival; GnRH-a, gonadotropin-releasing hormone agonist.

**Figure 1:**
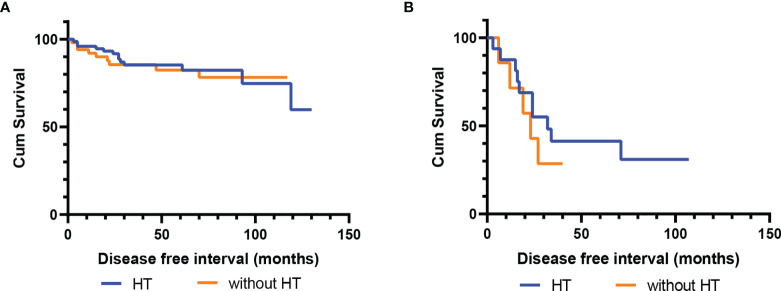
Disease-free survival (DFS) of patients with and without postoperative hormone therapy. **(A)** The cohort without fertility-sparing (p=0.802); **(B)** The fertility-sparing cohort (p=0.466).

The median follow-up time was 58 (6–135) months, and eight (6.2%) patients were lost to follow-up. A total of 22 (17.7%) patients had disease recurrence, and the median DFS was 47 (2–130) months; only one patient died as a result of LG-ESS recurrence. There was no statistically significant difference in recurrence between the groups with and without hormone therapy (p=0.802) (**Figure 1A**). Multivariate Cox regression analysis showed that ovarian preservation (p=0.004) and negative expression of ER/PR (p=0.000) were high-risk factors for recurrence, and HR was 6.250 (95% CI: 1.786-21.874) and 23.249 (95% CI: 4.912-110.026), respectively (**Table 2**). Subgroup analysis revealed that hormone therapy reduced the recurrence rate only in patients with stage II–IV disease (p=0.001, HR 0.144, 95% CI: 0.038-0.548) (**Supplementary Figure 1A** and **Table 3**); no significant difference was found in patients with stage I disease (p=0.256) (**Supplementary Figure 1B**). Hormone therapy appeared to prolong DFS in patients with ovarian preservation; however, this effect was not significant (p=0.331) (**Supplementary Figure 1C**). In patients with stage II–IV disease, high-dose progestins could notably reduce recurrence compared with no hormone therapy (p=0.012, HR 0.154, 95% CI: 0.036-0.660), whereas the non-progestin group was marginally better than the group without hormone therapy (p=0.054) (**Table 3**). Moreover, hormone therapy within 12 months was a protective factor against recurrence (p=0.038, HR 0.241, 95% CI: 0.063-0.922).

**Table 2:** Univariate and multivariate Cox regression of clinicopathological features for recurrence in the cohort without fertility-sparing.

Parameters	Univariable	Multivariable	
	P value	P value	HR (95% CI)
Age	0.010	0.316	0.970 (0.913-1.030)
BMI	0.401		
Menopausal status (premenopausal vs. postmenopausal)	0.175		
Ovarian preservation (yes vs. no)	0.000	0.004	6.250 (1.786-21.874)
Tumor diameter (>5cm vs. ≤5cm)	0.776		
LVSI (positive vs. negative)	0.101		
ER/PR staining (negative vs. positive)	0.014	0.000	23.249 (4.912-110.026)
Stage (II-IV vs. I)	0.123		
CA125 level (elevated vs. normal)	0.055	0.153	2.047 (0.766-5.468)
Radiotherapy (with vs. without)	0.253		
Chemotherapy (with vs. without)	0.356		
Hormone therapy (with vs. without)	0.803		

BMI, body mass index; LVSI, lymphovascular space involvement; ER, estrogen receptor; PR, progesterone receptor; HR, hazard ratio; CI, confidence interval.

**Table 3:** Univariate Cox regression of hormone therapy parameters in patients with stage II-IV disease.

Parameters	P value	HR (95% CI)
Hormone therapy (with vs. without)	0.005	0.144 (0.038-0.548)
Hormone therapy type		
High-dose progestins (with vs. no HT)	0.012	0.154 (0.036-0.660)
Non-progestins (with vs. no HT)	0.054	0.119 (0.014-1.037)
Hormone therapy duration		
≤12 months (with HT vs. no HT)	0.038	0.241 (0.063-0.922)
>12 months (with HT vs. no HT)	0.958	

HR, hazard ratio; CI, confidence interval; HT, hormone therapy.

### Outcomes in the Fertility-Sparing Cohort

**Table 4** summarizes the epidemiological characteristics, treatment, and follow-up results of the 23 patients in the fertility-sparing cohort. The median age and BMI were 29 (15–40) years and 22 (17.5-30.5) kg/m^2^, respectively. The most common clinical presentation was myoma (73.9%), followed by polypoid (21.7%); one patient had abdominal neoplasm. Resection of lesions by laparotomy was performed in 34.8%, by laparoscopy in 43.5%, and by hysteroscopy in 21.7% of patients. Almost all patients (95.7%) showed ER/PR expression. Twenty-two patients had stage I disease (7 with stage I A, and 12 with stage I B) and one patient had stage III B disease. In total, 16 patients (69.5%) received postoperative hormone therapy, including seven with only high-dose progestins and nine with non-progestin or combination therapy (all including GnRH-a). In terms of treatment duration, 11 patients (68.8%) received hormone therapy within 6 months, and five patients (31.2%) received over 6 months of therapy. None of these patients received chemotherapy after surgery.

**Table 4:** Epidemiological characteristics, treatment, and follow-up of the fertility-sparing cohort.

Parameters	Patients (n=23)
Age at diagnosis (years), median (range)	29 (15-40)
BMI at diagnosis (kg/m^2^), median (range)	22 (17.5-30.5)
Clinical presentation, n (%)	
Myoma	17 (73.9)
Polypoid	5 (21.7)
Myoma and abdominal neoplasm	1 (4.3)
Surgical method, n (%)	
Laparotomy	8 (34.8)
Laparoscopy	10 (43.5)
Hysteroscopy	5 (21.7)
Stage, n (%)	
I A	7 (30.4)
I B	12 (52.2)
I	3 (13.1)
III B	1 (4.3)
Immunohistochemical staining of ER/PR, n (%)	
Both positive	22 (95.7)
Not reported	1 (4.3)
Postoperative hormone therapy, n (%)	
High-dose Progestins	7 (30.4)
Non-progestin	9^*^ (39.1)
None	7 (30.4)
Duration of hormone therapy, n (%)	
≤6 months	11/16 (68.8)
>6 months	5/16 (31.2)
Chemotherapy, n (%)	0
Pregnancy outcomes, n (%)	
Delivery	6^#^ (26.1)
Ongoing pregnancy	1 (4.3)
Abortion	1 (4.3)
Recurrence after surgery, n (%)	15/23^※^ (65.2)
DFS (months), median (range)	24 (3-107)
Surgery after first recurrence, n (%)	
Hysterectomy	7 (46.7)
Lesion resection (fertility-sparing)	7^**^ (46.7)
No surgery	1 (6.6)
Hysterectomy finally performed after recurrences	12^##^/15 (80)
Follow-up time (months), median (range)	73 (19-121)
Loss to follow-up, n (%)	1 (4.3)

^*^Four patients were treated with GnRH-a, two with GnRH-a and LNG-IUD, two with GnRH-a and progestins, and one with GnRH-a and letrozole. ^#^Cesarean section was performed in 5 patients, and one patient had vaginal delivery. Hormone therapy was as follows: two patients received progestin, two received GnRH-a, one received GnRH-a and progestin, and one received no therapy. ^※^The patient experienced recurrence before she was lost to follow-up. ^**^The location of recurrence is extrauterine in three patients. ^##^Two patients gave birth before hysterectomy. BMI, body mass index; ER, estrogen receptor; PR, progesterone receptor; LVSI, lymphovascular space involvement; DFS, disease-free survival; GnRH-a, gonadotropin-releasing hormone agonist; LNG-IUD, levonorgestrel-releasing intrauterine device.

Twenty-two patients finished follow-up, with only one patient being lost; however, this patient had disease recurrence before being lost to follow-up. A total of 15 patients (65.2%) experienced recurrence of LG-ESS, and the median DFS was 24 (3–107) months; no patient died of LG-ESS. Among these patients, seven (46.7%) continued to preserve fertility after the first recurrence. Finally, 12 (80%) patients gave up preserving fertility and underwent hysterectomy after repeated recurrence; only two of these patients had given birth before surgery. Six patients had successfully delivered a healthy fetus (five with cesarean section and one with vaginal delivery), one patient had ongoing pregnancy, and one patient had an abortion. Among patients with successful delivery, only one did not receive hormone therapy; the remaining patients received high-dose progestins (n=2), GnRH-a (n=2), or progestins with GnRH-a (n=1). The median follow-up duration was 73 (19–121) months. Patients who received postoperative hormone therapy had a longer DFS, although this observation was not statistically significant (p=0.466) (**Figure 1B**).

## Discussion

LG-ESS is a rare gynecological malignancy that is generally diagnosed after surgery; thus, almost all studies are retrospective. To the best of our knowledge, this is the largest retrospective cohort study on the effect of hormone therapy on uterine LG-ESS recurrence. In particular, we analyzed patients with and without fertility-sparing.

### Cohort Without Fertility-Sparing

According to our results, the recurrence rate was 17.7% and the median DFS was 47 months in the cohort without fertility-sparing. Only one patient suffered rapid recurrence leading to death. Approximately 70% of patients had stage I disease. This confirmed that LG-ESS is a slow-progressing malignancy with late recurrence, and reasonably good prognosis (2, 15, 16).

More than half of the patients received postoperative hormone therapy, with the most common drug used being high-dose progestins (MA or MPA). Our study revealed that hormone therapy had no impact on recurrence in the entire cohort and in the stage I subgroup. However, in patients with stage II–IV disease, hormone therapy could significantly reduce recurrence. This inconsistency in outcomes between the whole and partial cohorts may be due to the low proportion of patients with stage II–IV disease. Malouf et al. found that adjuvant treatment including hormone therapy was associated with the absence of recurrence in patients with stages I-II ESS (17). A cohort study that enrolled 37 patients with LG-ESS reported that hormone therapy was associated with a lower recurrence rate, even in patients with stage I disease (14). Zhang et al. showed that hormone therapy was a protective factor in patients with LG-ESS, accompanied by improved progression-free survival (PFS) (18). In addition, we confirmed a high proportion (76.7%) of ER/PR expression, which is consistent with previous studies (19, 20). This may reflect the potential validity of hormone therapy in patients with LG-ESS (21). However, discrepancies regarding the role of hormone therapy do exist. A multicenter study reported that PFS was comparable between LG-ESS patients with and without adjuvant hormone therapy (22). Moreover, Zhou et al. reported that hormone therapy had no significant impact on DFS in patients with LG-ESS (16). A meta-analysis including 10 retrospective LG-ESS studies indicated that patients with hormone therapy showed a significantly lower risk of recurrence; nevertheless, hormone therapy had little benefit in reducing the recurrence risk in stage III–IV patients (23).

In patients with stage II–IV disease, high-dose progestins significantly decreased the risk of recurrence, whereas non-progestin therapy (letrozole or GnRH-a) had a marginal effect on reducing recurrence. This suggests that high-dose progestin should be the hormone therapy of preference in this context, but non-progestin hormonal treatments may also be effective when there are contraindications or intolerable side effects to progestin. Moreover, hormone therapy for less than a year in duration was sufficiently effective. Mizuno et al. supported MPA as a therapeutic option for residual or recurrent LG-ESS, even as first-line therapy (24). Reich et al. found a high percentage of aromatase expression positivity in patients with LG-ESS, implying that aromatase inhibitors (e.g., letrozole) may play a role in the treatment of LG-ESS (25). Another study comparing aromatase inhibitors with progestins for LG-ESS treatment indicated that aromatase inhibitors were superior to progestins because of longer recurrence-free survival and fewer side effects in patients with stage II–IV disease; this, however, is not consistent with our observations (5).

Our study revealed that ovarian preservation is a high-risk factor for recurrence in patients with LG-ESS. LG-ESS is considered a hormone-dependent tumor that commonly occurs in premenopausal patients, which account for over 90% of the patients reported in our study (16, 26). The ovary is the main organ producing estrogen and progesterone hormones in these patients; therefore, ovarian preservation carries a potential risk of recurrence. In addition, hormone therapy cannot completely inhibit ovarian endocrine function based on our analysis. A meta-analysis of 17 studies with 786 patients reported that the ovarian preservation group had a significantly higher recurrence rate than the bilateral salpingo-oophorectomy group (27). However, the benefits of bilateral oophorectomy are controversial. Karataşlı et al. and Li et al. both reported that ovarian preservation did not affect the recurrence of stage I LG-ESS (4, 15). Negative ER/PR expression was another risk factor for recurrence in our study. Conversely, Cade et al. found that ER/PR positivity was related to survival benefit but did not significantly affect recurrence-free survival in ESS (28). Zhou et al. also stated that the ER/PR status did not influence DFS in patients with LG-ESS (16). We noted that ER/PR staining was unknown in 20.2% of patients, because it was not reported in our hospital in earlier years. However, we still reported this part to make results more completely. We did not observe any other high-risk factors for recurrence, including FIGO stage, radiotherapy, chemotherapy, LVSI, and menopausal status, which is in line with some previous studies (14, 16). Further research is needed to explore the role of age and serum CA125 levels in disease recurrence.

### Fertility-Sparing Cohort

In the fertility-sparing cohort, the recurrence rate was 65.2% and the median DFS was 24 months. The prognosis was evidently worse than that in the cohort without fertility-sparing, even though almost all patients had stage I disease and expressed ER/PR. Most patients underwent lesion resection by laparoscopy or laparotomy, possibly because myoma was the most common clinical presentation.

The proportion of patients receiving postoperative hormone therapy was 69.5% in our study, whereas it was as high as 83.3%-100% in other studies (29–31). However, our cohort included a larger number of patients compared to these studies. Except for high-dose progestins, GnRH-a was a relatively common choice of hormone therapy in the fertility-sparing cohort; this was quite different from the cohort without fertility-sparing. The most common duration of hormone therapy was 6 months or less, which was shorter than that described in other studies (30–32). Hormone therapy seemed to prolong DFS in these patients, although this effect was not significant. Encouragingly, six patients successfully delivered healthy fetuses after fertility-sparing management. Among these patients, five received hormone therapy, including high-dose progestins and/or GnRH-a. In the few reports so far, patients with successful delivery used only progestins for hormone therapy (29–32). Thus, our findings suggest that GnRH-a is also a good option for fertility-sparing hormone therapy. However, we observed that nearly half of the patients chose hysterectomy after the first recurrence, and this proportion eventually reached 80% after repeated recurrence. Only two of those patients had given birth before hysterectomy. This reflects that the pregnancy outcomes of fertility-sparing patients are not optimistic; therefore, patients should be fully informed of the risks and make careful choices.

Our study has several limitations that need to be mentioned. First, it was a retrospective study conducted in a single-center. However, despite this, our study is still convincing because of the relatively large number of enrolled patients, relatively long follow-up times, and low rate of loss to follow-up. Second, the different types, doses, and durations of hormone therapy, may have interfered with the overall impact of hormone therapy on recurrence.

In conclusion, LG-ESS is an indolent malignancy with a generally good survival outcome. In patients without fertility-sparing, postoperative hormone therapy reduced recurrence in stage II–IV but not in stage I disease. Moreover, no more than one year of high-dose progestins is recommended in patients with stage II–IV disease. Ovarian preservation and negative ER/PR expression are high-risk factors for recurrence in patients without fertility-sparing. Patients with fertility-sparing have a high risk of recurrence and poor pregnancy outcomes; therefore, they should be fully informed of these risks. For patients that have intense fertility desire and choose fertility-sparing surgery, postoperative hormone therapy (high-dose progestins and/or GnRH-a) may be a reasonable choice because it showed the tendency to prolong DFS.

## Data Availability Statement

The data underlying this article will be shared by the corresponding author, upon reasonable request. Requests to access these datasets should be directed to PP, pengpeng@pumch.cn.

## Ethics Statement

The studies involving human participants were reviewed and approved by The Institutional Review Board of Peking Union Medical College Hospital. Written informed consent for participation was not required for this study in accordance with the national legislation and the institutional requirements.

## Author Contributions

PP contributed to conception and design of the study. XH organized the database, performed the statistical analysis, and wrote the first draft of the manuscript. All authors contributed to manuscript revision, read, and approved the submitted version.

## Conflict of Interest

The authors declare that the research was conducted in the absence of any commercial or financial relationships that could be construed as a potential conflict of interest.

## Publisher’s Note

All claims expressed in this article are solely those of the authors and do not necessarily represent those of their affiliated organizations, or those of the publisher, the editors and the reviewers. Any product that may be evaluated in this article, or claim that may be made by its manufacturer, is not guaranteed or endorsed by the publisher.

## References

[1] Tropé CG Abeler VM Kristensen GB. Diagnosis and Treatment of Sarcoma of the Uterus. A Review. Acta Oncol (2012) 51(6):694–705. doi: 10.3109/0284186x.2012.689111 22793037

[2] Amant F Floquet A Friedlander M Kristensen G Mahner S Nam EJ. Gynecologic Cancer Intergroup (Gcig) Consensus Review for Endometrial Stromal Sarcoma. Int J Gynecol Cancer (2014) 24(9 Suppl 3):S67–72. doi: 10.1097/igc.0000000000000205 25033257

[3] Conklin CM Longacre TA. Endometrial Stromal Tumors: The New Who Classification. Adv Anat Pathol (2014) 21(6):383–93. doi: 10.1097/pap.0000000000000046 25299308

[4] Li AJ Giuntoli RL 2nd Drake R Byun SY Rojas F Barbuto D. Ovarian Preservation in Stage I Low-Grade Endometrial Stromal Sarcomas. Obstet Gynecol (2005) 106(6):1304–8. doi: 10.1097/01.AOG.0000185511.91694.1e 16319256

[5] Deshmukh U Black J Perez-Irizarry J Passarelli R Levy K Rostkowski A. Adjuvant Hormonal Therapy for Low-Grade Endometrial Stromal Sarcoma. Reprod Sci (2019) 26(5):600–8. doi: 10.1177/1933719118778801 29843577

[6] Nasioudis D Mastroyannis SA Latif NA Ko EM Haggerty AF Kim SH. Effect of Bilateral Salpingo-Oophorectomy on the Overall Survival of Premenopausal Patients With Stage I Low-Grade Endometrial Stromal Sarcoma; a National Cancer Database Analysis. Gynecol Oncol (2020) 157(3):634–8. doi: 10.1016/j.ygyno.2020.04.001 32354469

[7] Chang KL Crabtree GS Lim-Tan SK Kempson RL Hendrickson MR. Primary Uterine Endometrial Stromal Neoplasms. A Clinicopathologic Study of 117 Cases. Am J Surg Pathol (1990) 14(5):415–38. doi: 10.1097/00000478-199005000-00002 2327549

[8] Lim MC Lee S Seo SS. Megestrol Acetate Therapy for Advanced Low-Grade Endometrial Stromal Sarcoma. Onkologie (2010) 33(5):260–2. doi: 10.1159/000305661 20502061

[9] Altal OF Al Sharie AH Halalsheh OM Tashtush N Shaban S Alfaqih M. Complete Remission of Advanced Low-Grade Endometrial Stromal Sarcoma After Aromatase Inhibitor Therapy: A Case Report. J Med Case Rep (2021) 15(1):262. doi: 10.1186/s13256-021-02838-x 33947445 PMC8097811

[10] Burke C Hickey K. Treatment of Endometrial Stromal Sarcoma With a Gonadotropin-Releasing Hormone Analogue. Obstet Gynecol (2004) 104(5 Pt 2):1182–4. doi: 10.1097/01.AOG.0000133533.05148.aa 15516445

[11] Maccaroni E Lunerti V Agostinelli V Giampieri R Zepponi L Pagliacci A. New Insights Into Hormonal Therapies in Uterine Sarcomas. Cancers (Basel) (2022) 14(4):921. doi: 10.3390/cancers14040921 35205669 PMC8870116

[12] Banno K Kisu I Yanokura M Tsuji K Masuda K Ueki A. Progestin Therapy for Endometrial Cancer: The Potential of Fourth-Generation Progestin (Review). Int J Oncol (2012) 40(6):1755–62. doi: 10.3892/ijo.2012.1384 22366992

[13] Thanopoulou E Judson I. Hormonal Therapy in Gynecological Sarcomas. Expert Rev Anticancer Ther (2012) 12(7):885–94. doi: 10.1586/era.12.74 22845404

[14] Comert GK Turkmen O Kar I Yucel O Kilic C Boran N. Hormone Therapy Following Surgery in Low-Grade Endometrial Stromal Sarcoma: Is It Related to a Decrease in Recurrence Rate? J Chin Med Assoc (2019) 82(5):385–9. doi: 10.1097/JCMA.0000000000000039 31058712

[15] Karataşlı V Çakır İ Can B Erkılınç S Karadeniz T Kuru O. Does Ovarian Preservation Have an Effect on Recurrence of Early Stage Low-Grade Endometrial Stromal Sarcoma? J Obstet Gynaecol (2021) 41(5):797–802. doi: 10.1080/01443615.2020.1803238 33063586

[16] Zhou J Zheng H Wu SG He ZY Li FY Su GQ. Influence of Different Treatment Modalities on Survival of Patients With Low-Grade Endometrial Stromal Sarcoma: A Retrospective Cohort Study. Int J Surg (2015) 23(Pt A):147–51. doi: 10.1016/j.ijsu.2015.09.072 26449652

[17] Malouf GG Duclos J Rey A Duvillard P Lazar V Haie-Meder C. Impact of Adjuvant Treatment Modalities on the Management of Patients With Stages I-II Endometrial Stromal Sarcoma. Ann Oncol (2010) 21(10):2102–6. doi: 10.1093/annonc/mdq064 20305035

[18] Zhang Y Li Y Huang H Yang J Wu M Jin Y. Low-Grade Endometrial Stromal Sarcoma and Uterine Adenosarcoma: A Comparison of Clinical Manifestations and Outcomes. J Cancer (2019) 10(15):3352–60. doi: 10.7150/jca.30691 PMC6603426 31293638

[19] Reich O Regauer S Urdl W Lahousen M Winter R. Expression of Oestrogen and Progesterone Receptors in Low-Grade Endometrial Stromal Sarcomas. Br J Cancer (2000) 82(5):1030–4. doi: 10.1054/bjoc.1999.1038 PMC2374426 10737385

[20] Cui R Yuan F Wang Y Li X Zhang Z Bai H. Clinicopathological Characteristics and Treatment Strategies for Patients With Low-Grade Endometrial Stromal Sarcoma. Med (Baltimore) (2017) 96(15):e6584. doi: 10.1097/md.0000000000006584 PMC5403086 28403089

[21] Katz L Merino MJ Sakamoto H Schwartz PE. Endometrial Stromal Sarcoma: A Clinicopathologic Study of 11 Cases With Determination of Estrogen and Progestin Receptor Levels in Three Tumors. Gynecol Oncol (1987) 26(1):87–97. doi: 10.1016/0090-8258(87)90074-6 3792939

[22] Stewart LE Beck TL Giannakopoulos NV Rendi MH Isacson C Goff BA. Impact of Oophorectomy and Hormone Suppression in Low Grade Endometrial Stromal Sarcoma: A Multicenter Review. Gynecol Oncol (2018) 149(2):297–300. doi: 10.1016/j.ygyno.2018.03.008 29534832

[23] Cui R Cao G Bai H Zhang Z. The Clinical Benefits of Hormonal Treatment for Lg-Ess: A Meta-Analysis. Arch Gynecol Obstet (2019) 300(5):1167–75. doi: 10.1007/s00404-019-05308-4 31583462

[24] Mizuno M Yatabe Y Nawa A Nakanishi T. Long-Term Medroxyprogesterone Acetate Therapy for Low-Grade Endometrial Stromal Sarcoma. Int J Clin Oncol (2012) 17(4):348–54. doi: 10.1007/s10147-011-0299-y 21830086

[25] Reich O Regauer S. Aromatase Expression in Low-Grade Endometrial Stromal Sarcomas: An Immunohistochemical Study. Mod Pathol (2004) 17(1):104–8. doi: 10.1038/sj.modpathol.3800031 14631363

[26] Cheng X Yang G Schmeler KM Coleman RL Tu X Liu J. Recurrence Patterns and Prognosis of Endometrial Stromal Sarcoma and the Potential of Tyrosine Kinase-Inhibiting Therapy. Gynecol Oncol (2011) 121(2):323–7. doi: 10.1016/j.ygyno.2010.12.360 21277011

[27] Nasioudis D Ko EM Kolovos G Vagios S Kalliouris D Giuntoli RL. Ovarian Preservation for Low-Grade Endometrial Stromal Sarcoma: A Systematic Review of the Literature and Meta-Analysis. Int J Gynecol Cancer (2019) 29(1):126–32. doi: 10.1136/ijgc-2018-000063 30640694

[28] Cade TJ Quinn MA Rome RM Polyakov A. Prognostic Significance of Steroid Receptor Positivity and Adjuvant Progestogen Use in Endometrial Stromal Sarcoma. Aust N Z J Obstet Gynaecol (2014) 54(5):453–6. doi: 10.1111/ajo.12245 25287561

[29] Xie W Cao D Yang J Jiang X Shen K Pan L. Fertility-Sparing Surgery for Patients With Low-Grade Endometrial Stromal Sarcoma. Oncotarget (2017) 8(6):10602–8. doi: 10.18632/oncotarget.12491 PMC5354684 27736798

[30] Laurelli G Falcone F Scaffa C Messalli EM Del Giudice M Losito S. Fertility-Sparing Management of Low-Grade Endometrial Stromal Sarcoma: Analysis of an Institutional Series and Review of the Literature. Eur J Obstet Gynecol Reprod Biol (2015) 195:61–6. doi: 10.1016/j.ejogrb.2015.09.041 26476800

[31] Zheng Y Yin Q Yang X Dong R. Fertility-Sparing Management of Low-Grade Endometrial Stromal Sarcoma: Analysis of an Institutional Series, a Population-Based Analysis and Review of the Literature. Ann Transl Med (2020) 8(21):1358. doi: 10.21037/atm-20-2180 33313103 PMC7723593

[32] Dong R Pang Y Mao H Yang N Liu P. Successful Pregnancy Following Conservative Management of Low-Grade Endometrial Stromal Sarcoma: A Case Report. Oncol Lett (2014) 7(4):1039–42. doi: 10.3892/ol.2014.1858 PMC3961328 24944665

