# Supplementary material for: Hormone Therapy Reduces Recurrence in Stage II-IV Uterine Low-Grade Endometrial Stromal Sarcomas: A Retrospective Cohort Study
Source: Front Oncol. 2022 Jun 28;12:922757. doi: 10.3389/fonc.2022.922757 (PMC9275776; doi:10.3389/fonc.2022.922757)
Supplement: Supplementary file 1 [file Image_1.pdf]

Supplementary Material

1 Supplementary Figures and Tables

1.1 Supplementary Figures

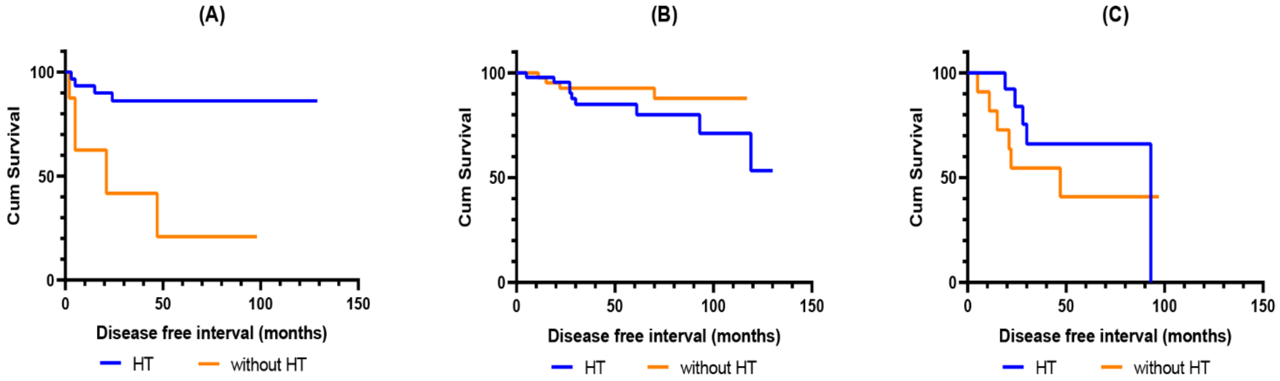

Supplementary Figure 1. Disease-free survival (DFS) of patients with and without postoperative hormone therapy in different subgroups without fertility-sparing. (A) Stage II-IV disease group (p=0.001); (B) Stage I disease group (p=0.256); (C) Ovarian preservation group (p=0.331).
